# Reduced levels of plasma polyunsaturated fatty acids and serum carnitine in autistic children: relation to gastrointestinal manifestations

**DOI:** 10.1186/s12993-014-0048-2

**Published:** 2015-02-07

**Authors:** Gehan A Mostafa, Laila Y AL-Ayadhi

**Affiliations:** Department of Pediatrics, Faculty of Medicine, Ain Shams University, Address: 9 Ahmed El-Samman Street off Makram Ebaid, Nasr City, Cairo Egypt; Autism Research and Treatment Center, AL-Amodi Autism Research Chair, Department of Physiology, Faculty of Medicine, King Saud University, Riyadh, Saudi Arabia

**Keywords:** Autism, Brain energy, Carnitine, Docosahexaenoic acid, Gastrointestinal manifestations, Polyunsaturated fatty acids

## Abstract

**Background:**

Gastrointestinal (GI) manifestations are common in autistic children. Polyunsaturated fatty acids (PUFAs) and carnitine are anti-inflammatory molecules and their deficiency may result in GI inflammation. The relationship between the increased frequency of GI manifestations and reduced levels of PUFAs and carnitine was not previously investigated in autistic patients. This study was the first to investigate plasma levels of PUFAs and serum carnitine in relation to GI manifestations in autistic children.

**Methods:**

Plasma levels of PUFAs (including linoleic, alphalinolenic, arachidonic “AA” and docosahexaenoic “DHA” acids) and serum carnitine were measured in 100 autistic children and 100 healthy-matched children.

**Results:**

Reduced levels of serum carnitine and plasma DHA, AA, linolenic and linoleic acids were found in 66%, 62%, 60%, 43% and 38%, respectively of autistic children. On the other hand, 54% of autistic patients had elevated ω6/ω3 ratio. Autistic patients with GI manifestations (48%) had significantly decreased levels of serum carnitine and plasma DHA than patients without such manifestations. In addition, autistic patients with GI manifestations had significantly increased percentage of reduced serum carnitine (91.7%) and plasma DHA levels (87.5%) than patients without such manifestations (42.3% and 38.5%, respectively), (P < 0.001 and P < 0.001%, respectively).

**Conclusions:**

Reduced levels of plasma DHA and serum carnitine levels may be associated with the GI problems in some autistic patients. However, this is an initial report, studies are recommended to invesigate whether reduced levels of carnitine and DHA are a mere association or have a pathogenic role in GI problems in autistic patients.

## Background

Some cases of autism may be associated with several organic conditions including disturbance of brain energy metabolism which depends on adequate supply of polyunsaturated fatty acids (PUFAs) and carnitine and normal mitochondrial function [1,2]. Fatty acids are key nutrients that affect early growth and development. Among the fatty acids, omega-3 polyunsaturated fatty acids (n-3 PUFA) and omega-6 PUFA (n-6 PUFA) which have been suggested to decrease and increase the severity of several human diseases, respectively. PUFA are long-chained and unsaturated molecules only obtained by dietary intake of certain grains such as flaxseed and canola, walnuts, and sea fish. PUFA that contain more than one carbon- carbon double bond are classified into n-6 and n-3 fatty acids that have, in common, a final carbon-carbon double bond in the n-3 (n-3 PUFA) or n-6 (n-3 PUFA) position, counting from the methyl end. The numeric designations used for PUFA is derived from the number of carbon atoms, followed by the number of sites of unsaturation. Linoleic acid (LA) is the shortest-chained omega − 6 fatty acid serving as a substrate that can be converted to arachidonic acid (AA) which is an important omega − 6 fatty as it is the precursor for prostaglandins [3]. α-linolenic acid (ALA), eicosapentaenoic acid (EPA) and docosahexaenoic acid (DHA) are important n-3 PUFA contributing to either achieving optimal health or protection against diseases, and even longevity. Human brain is 60% fat of which 25% is DHA [4,5].

The optimal range of the ratio of omega6/omega3 (AA/DHA) varies from 1/1 to 4/1. High ω6/ω3 ratio promotes the pathogenesis of many diseases. Whereas low ω6/ω3 ratio exerts a suppressive effect. A ratio of ω6/ω3 of 4/1 is the optimal ratio of brain mediated function [6]. PUFAs are needed for normal brain development and function as PUFAs modulate the function of many neurotransmitters, membrane fluidity, intracellular Ca(2+) signaling and hence function of the neuronal cell [5]. The role of (n-3) fatty acids in controlling inflammation and protecting neuron cells from oxidative damage has been reported, so they could be beneficial in treatment of some psychiatric illnesses [7]. Carnitine, a nutrient synthesized in the liver and kidney, is essential for transport of long chain fatty acids across inner mitochondrial membrane for β- oxidation and energy production. Synaptic transmission of multiple neurotransmitters needs the neurobiological effect of acetyl carnitine [8].

Gastrointestinal (GI) disturbances such as abdominal pain, chronic diarrhea, constipation, vomiting, gastroesophageal reflux, and intestinal infections.are commonly reported in children with autism, and may contribute to behavioral impairment [9,10]. Reports of deficiencies in disaccharidase enzymatic activity and of beneficial responses to probiotic and dietary therapies led researchers to survey gene expression and the mucoepithelial microbiota in intestinal biopsies from children with autism. Autistic children can benefit from adaptation of general pediatric guidelines for the diagnostic evaluation of abdominal pain, chronic constipation, and gastroesophageal reflux disease [11]. Carnitine and PUFAs are anti-inflammatory molecules and their deficiency may result in GI inflammation and gut injury [12,13]. Patients with chronic intestinal disease should be evaluated for likely PUFAs deficiencies and imbalances, and treated with substantial amounts of supplements rich in PUFAs, such as oral vegetable and fish oils, or intravenous lipids if necessary [14].

The relationship between the increased frequency of GI manifestations and reduced levels of PUFAs and carnitine was not previously investigated in autistic patients. This study was the first to investigate plasma levels of PUFAs and serum carnitine in relation to GI manifestations in autistic children. This may add new biological indices and therapeutic implications for autism.

## Methods

### Study population

This cross-sectional study was conducted on 100 children with autism. They were recruited from the Autism Research and Treatment Center, Faculty of Medicine, King Saud University, Riyadh, Saudi Arabia. Patients were fulfilling the criteria of the diagnosis of autism according to the 4th edition of the Diagnostic and Statistical Manual of Mental Disorders [15]. The autistic group comprised 78 males and 22 females. Their ages ranged between 3 and 10 years (mean ± SD = 6.22 ± 2.1 years). Patients who had associated neurological diseases (such as cerebral palsy and tuberous sclerosis) and metabolic disorders (eg. Phenylketonuria) were excluded form the study. Also, autistic patients on PUFAs or anticonvulsants therapy were not included.

The control group comprised 100 age- and sex- matched apparently healthy children They included 78 males and 22 females. They were the healthy older siblings of the healthy infants who attend the Well Baby Clinic, King Khalid University Hospital, Faculty of Medicine, King Saud University, Riyadh, Saudi Arabia for routine following-up of their growth parameters. The control children were not related to the children with autism, and demonstrated no clinical findings suggestive of immunological, GI or neuropsychiatric disorders. Their ages ranged between 3 and 10 years (mean ± SD = 5.96 ± 2 years).

None of the studied subjects was vegetarian or was on a restricted diet such as casein and guten-free diet. All studied subjects had normal body weight (body mass index (BMI) was between the fifth and less than the 85th percentiles based on age and sex). Also, none of the studied subjects had abnormal results of stool analysis.

The local Ethical Committee of the Faculty of Medicine, King Saud University, Riyadh, Saudi Arabia, approved this study. In addition, an informed written consent of participation in the study was signed by the parents or the legal guardians of the studied subjects.

### Study measurements

#### Clinical evaluation of autistic patients

This was based on clinical history taking from caregivers, clinical examination and neuropsychiatric assessment. Gastrointestinal symptoms were assessed with the parent-report version of the Questionnaire on Pediatric Gastrointestinal Symptoms by an experienced pediatric gastroenterologist according to the Questionnaire on Pediatric Gastrointestinal Symptoms - Rome III Version used by previous studies that assessed gastrointestinal dysfunction in autism [16]. In addition, stool analysis was done for all patients.

The degree of the disease severity was assessed by using the Childhood Autism Rating Scale (CARS) [17] which rates the child on a scale from one to four in each of fifteen areas (relating to people; emotional response; imitation; body use; object use; listening response; fear or nervousness; verbal communication; non-verbal communication; activity level; level and consistency of intellectual response; adaptation to change; visual response; taste, smell and touch response and general impressions). According to the scale, children who have scored 30–36 have mild to moderate autism (n = 38), while those with scores ranging between 37 and 60 points have a severe degree of autism (n = 62).

#### Samples collection

Blood samples were collected in the morning following at least 10 hour period of fasting. Blood samples were equally subdivided into two clean tubes, the first tube was dry and the blood was left to clot, then the serum was separated and stored at −20°C until assay of carnitine. The second tube was containing fluoride EDTA as an anticoagulant, then centrifugation of the samples was done immediately and plasma was separated and stored in sterile aliquoutes at −80°C until assay of PUFAs.

#### Assessment of serum carnitine

Serum carnitine was measured by using enzymatic ultraviolet test from Roche Diagnostics Gmbh, Cat. No. 1′ 242008, Germany [18]. To increase accuracy, all samples were analyzed twice in two independent experiments to assess inter-assay variations and to ensure reproducibility of the observed results (P > 0.05). No significant cross-reactivity or interference was observed.

#### Determination of plasma polyunsaturated fatty acids (Linoleic, Linolenic, Arachidonic and Docosahexaeinoic acids)

The principle was the injection of separated fatty acids from the plasma into the gas chromatography instrument. Separation of fatty acids from plasma was done by addition of 3 ml of 3 g/L solution of nonanoic acid in 100 μL of acetyl chloride slowly with magnetic strirring for 45 min at room temperature. Three ml of 6% potassium carbonate solution was added in water with stiring while cooling the mixture in an ice bath. After that, 300 μL of hexan and vortex were added and the sample was cooled at 4°c for 30 minutes. Then, centrifugation was done for 10 minutes. Finally, 100 μ of the upper layer was removed and 1.1 μL was subjected to Gas Chromatography H5890 (Hewlett-Pakard, Palo Alto, CA) [19]. To increase accuracy, all samples were analyzed twice in two independent experiments to assess inter-assay variations and to ensure reproducibility of the observed results (P > 0.05). No significant cross-reactivity or interference was observed.

### Statistical analysis

The results were analyzed by the commercially available software package (Statview, Abacus concepts, inc., Berkley, CA). The data was non-parametric and was presented as median and interquartile range (IQR), which is the difference between the 75th and 25th percentiles. Mann–Whitney *U*-test was used for comparison of data. Chi-square test was used for comparison between qualitative variables of the studied groups. Spearman’s rho correlation coefficient “r” was used to determine the relationship between different variables. For all tests, a probability (P) of less than 0.05 was considered significant. As data distribution was non-parametric, patients were considered to have decreased serum carnitine, plasma DHA, linolenic, arachidonic and linoleic acids if their levels were below the 5th percentile of the control values (4.96 mg/ml, 1.35 μg/ml, 1.56 μg/ml, 2.64 μg/ml, and 1.38 μg/ml respectively). On the other hand, patients were considered to have elevated ω6/ω3 ratio if their levels were above the 95th percentile of the control values (3.84).

## Results

### Plasma PUFAs and serum carnitine levels in autistic children

Autistic patients had significantly lower levels of plasma PUFAs and serum carnitine than healthy children. On the other hand, ω6/w3 ratio (AA/DHA) was significantly higher in autistic patients than healthy children (Table 1). Low serum carnitine and plasma DHA, AA, linolenic and linoleic acids, below the 5th percentile of the control values, were found in 66%, 62%, 60%, 43% and 38%, respectively of autistic children. On the other hand, 54% of autistic patients had elevated ω6/ω3 ratio above the 95th percentile of the control values.

**Table 1:** **Plasma PUFAs and serum carnitine levels in autistic patients and healthy control children**

	**Autistic patients (n = 100) Median (IQR)**	**Healthy controls (n = 100) Median (IQR)**	**P-value**
Serum carnitine (mg/ml)	3.3 (2)	7.2 (2.6)	< 0.001
Plasma DHA (ω3) (μg/ml)	0.18 (1.2)	3.1 (0.8)	< 0.001
Plasma linolenic (μg/ml)	1.6 (1.3)	3.3 (2.6)	<0.01
Plasma AA (ω6) (μg/ml)	1.7 (1.8)	4.2 (3.4)	<0.01
Plasma linoleic (μg/ml)	1.3 (2.6)	3.2 (1.5)	<0.05
ω6/ω3	3 (2.4)	1.8 (1.4)	<0.01

There was a significant positive association between the reduced serum levels of carnitine and the elevated ω6/w3 ratio in autistic children as the percentage of elevated ω6/w3 was significantly higher in autistic patients with reduced serum levels of carnitine (94.7%) than patients with normal serum carnitine levels (48.4%)), P < 0.001.

Plasma levels of PUFAs and serum levels of carnitine had no significant correlations with CARS and the age of autistic children (P > 0.05). Also, plasma levels of PUFAs did not correlated significantly with serum carnitine levels (P > 0.05).

### Relationship between gastrointestinal manifestations and both plasma PUFAs and serum carnitine levels in autistic children

Gastrointestinal manifestations were found in 48 autistic children (48%). They include recurrent unexplained attacks of epigastric pain and chromic constipation in 20 patients, chronic constipation and bloating in 22 patients and recurrent attacks of diarrhea, with normal results of stool analysis, in 6 patients. These symptoms were reported by the parents of autistic children. They were recurrent, severe and the patients were attending the clinic because of these agonizing symptoms. None of the autistic children had abnormal results of stool analysis.

Autistic patients with GI manifestations had significantly lower serum carnitine and plasma DHA than patients without such manifestations. On the other hand, ω6/w3 ratio was significantly higher in autistic patients with GI problems than patients without such manifestations (Table 2).

**Table 2:** **Plasma PUFAs and serum carnitine levels in autistic patients with and without GI manifestations**

	**Autistic patients with GI manifestations (n = 48) Median (IQR)**	**Autistic patients without GI manifestations (n = 52) Median (IQR)**	**P-value**
Serum carnitine (mg/ml)	2.3 (2.2)	5 (3)	< 0.001
Plasma DHA (ω3) (μg/ml)	0.12 (1)	2.2 (0.9)	< 0.001
Plasma linolenic (μg/ml)	1.2 (1.3)	2 (2)	>0.05
Plasma AA (ω6) (μg/ml)	1.1 (1.6)	1.8 (3.4)	>0.05
Plasma linoleic (μg/ml)	1.2 (3)	2 (2.5)	>0.05
ω6/ω3	4.8 (1.6)	2.5 (1.4)	<0.01

Autistic patients with GI manifestations had significantly higher percentage of reduced serum carnitine (91.7%) and plasma DHA levels (87.5%) than patients without such manifestations (42.3% and 38.5%, respectively), (P < 0.001 and P < 0.001%, respectively). On the other hand, autistic patients with GI manifestations had significantly higher percentage of elevated ω6/w3 ratio (62.5%) than patients without such manifestations (15.4%), P < 0.001.

## Discussion

In the current study, autistic patients had significantly lower levels of plasma PUFAs than healthy children. On the other hand, ω6/w3 ratio (AA/DHA) was significantly higher in autistic patients than healthy children. One preliminary study found that average total levels of omega-3 (n-3 PUFAs) in the autistic children were about 20% lower than mentally retarded children used as controls. Levels of DHA were 23% lower. These deficiencies resulted in a significantly higher ratio of n-6 to n-3 in 25% of autistic children [20]. It is widely accepted that the PUFAs have an important role in many neural pathways and that their deficiency may be correlated with the occurrence of several psychiatric illnesses such as major depression, attention-deficit hyperactivity disorders, dyslexia, dyspraxia and autism and they are featured as adjunctive or as monotherapy for treatment of these diseases with many surprising results [7,21,22]. Although no definite conclusions can be drawn on the therapeutic efficacy of omegs-3 PUFAs in some psychiatric diseases, the evidence suggests that these molecules have a potential preventive role in people at extremely high risk for developing psychosis [23].

The mechanisms by which DHA can impact on neuronal functions involve the modulation of membrane biophysical properties, the regulation of neurotransmitter release (such as dopamine and serotonin), the synthesis of oxygenated biologically active derivatives, and the nuclear receptor mediated transcription of lipid responsive genes [24]*.* Some studies of human infants suggest that dietary DHA may play a role in cognitive development and in some neurodevelopmental disorders like autism [25]. Placebo-based studies established the appropriate therapeutic dose of (n-3) fatty acids, varying from the recommended dietary dose to an amount that may be 3 or 4 times higher. Amminger et al. [26] conducted a pilot trial with 13 autistic children for 6 wk in which 1.5 g (n-3) fatty acid composition were given. They observed a statistically significant change in hyperactivity and stereotypy of behavior in autistic children compared with the placebo group. On the other hand, the effect of omega-3 FA supplementation in adults with autism may not be beneficial as one study reported no significant improvement of behavioral abnormalities after omega-3 FA supplementation in adults with severe autism [27].

The current study revealed lower levels of serum carnitine in autistic patients than healthy children. A previous study also reported carnitine deficiency in a group of autistic children. Acetyl carnitine is needed for mitochondrial transfere of PUFAs and synaptic transmission of multiple neurotransmitters which affect attention, antagonize deterioration of ability to learn and improve long term memory. Carnitine has been proposed to be a conditionally essential nutrient, and even termed vitamin B_T_. Carnitine homeostasis in humans is maintained by a modest rate of endogenous synthesis, absorption from dietary sources, and efficient tubular reabsorption by the kidney [28]. Carnitine deficiency can develop secondary to dietary inadequacy or as an adverse effect of medical treatment secondary to administration of pivalate-conjugated antibiotics or valproic acid [29]. A deletion of the 6-N-trimethyllysine dioxygenase gene, the first enzyme of the carnitine biosynthesis pathway in mitochondria, was discovered while studying probands with autism [30]. Mitochondrial defect which is the most common metabolic abnormality associated with autism may be the origin of carnitine deficiency in some autistic children. Several studies suggested that nutritional supplements may be beneficial in some children with autism who have mitochondrial dysfunction or abnormal biomarkers of mitochondrial function. Carnitine was the most commonly noted supplement to be helpful [31,32].

Anhedonia is a condition in which the capacity of experiencing pleasure is totally or partially lost and it was reported in various psychiatric disorders [33]. Selective social anhedonia was reported in autism [34]. The use of acetyl-l-carnitine to treat anhedonia, melancholic, and negative symptoms in anhedonic alcoholics was proposed [35] due to the effect of acetyl-l-carnitine on dopamine outflow, which is mostly implicated in anhedonia, alcohol and substance use disorders. Carnitine supplementation has been shown to significantly increase the levels of dopamine in the cortex, hippocampus, and striatum of rat brain [36]. Martinotti et al. (2011) reported the efficacy and safety of acetyl-l-carnitine in the treatment of anhedonia, melancholia, and negative symptoms in anhedonic alcoholics after 10 days of intravenous therapy [37]. Acetyl-l-carnitine was also reported to be effective in the treatment of non-alcoholic fatty liver disease [38]. L-carnitine therapy (50 mg/kilogram-bodyweight/day) administered for 3-months significantly improved several clinical measurements of ASD severity [1], but subsequent studies are recommended.

In the present work, GI manifestations (recurrent unexplained attacks of epigastric pain, chromic constipation, bloating and recurrent attacks of diarrhea, with normal results of stool analysis) were found in 48%.of autistic patients. The reported prevalence of GI abnormalities in autism is high [11]. A previous research reported that the majority (61%) of children had at least one reported gastrointestinal symptom [39]. In this study, 91.7% of autistic patients with GI manifestations had chronic constipation which was reported to be the most common type of GI dysfunction in children with autism (85%). As children with autism can benefit from adaptation of general pediatric guidelines for the diagnostic evaluation of GI problems [11], this should be addressed in future studies. In addition, this study depended on clinical manifestations and stool analysis for detection of gastrointestinal dysfunction in autistic children Thus, investigations such as stool bacteriology, GI endoscopy and biopsy should be performed in future studies for better evaluation of GI problems in autistic children.

How GI factors are related to autism is not yet clear. Many patients with autism have a history of previous antibiotic exposure, GI symptoms, abnormal food cravings, and unique intestinal bacterial populations, which have been proposed to relate to variable symptom severity [40]. Recent evidence suggests potential, but unproven, links between dietary, metabolic, infective, and gastrointestinal factors and the behavioral exacerbations and remissions of autism. Propionic acid and its related short-chain fatty acids (SCFAs) are fermentation products of autism-associated bacteria (Clostridia, Bacteriodetes, Desulfovibrio). SCFAs represent a group of compounds derived from the host microbiome that are plausibly linked to autism and can induce widespread effects on gut, brain, and behavior [41]. The strong correlation of GI symptoms with autism severity indicates that children with more severe autism are likely to have more severe gastrointestinal symptoms and vice versa. The low level of SCFA’s was partly associated with increased probiotic use, and probably due to either lower production and/or greater absorption. Percentage DNA damage, tail length, and tail moment are adequate biomarkers of propionic acid neurotoxicity due to oral administration or as a metabolite of induced enteric bacterial overgrowth [42].

In this work, autistic patients with GI manifestations had significantly lower serum carnitine and plasma DHA than patients without such manifestations. These results may demonstrate the possible association between GI manifestations and the reduced levels of serum carnitine and plasma DHA in autistic patients. The relationship between GI manifestations and plasma levels of PUFAs and serum carnitine in autistic children was not previously investigated.

All varieties of (n-3) acids (mainly alpha-linoleic, DHA, and EPA) are essential components in mammalian metabolism, whether they act as anti-inflammatory molecules, immune-modulating agents, and the main components in guaranteeing cell membrane stability [12]. PUFAs are known to modulate inflammation. Preoperative parenteral PUFA-LE supplementation, preferably by marine (n-3) PUFA, ameliorates postoperative intestinal inflammation and dysmotility [43]. Carnitine’s role in the GI tract has recently been highlighted by several publications linking mutations in genes encoding carnitine transporters OCTN1 (SLC22A4) and OCTN2 (SLC22A5) with Crohn’s disease [44]. Carnitine plays a major role in neonatal mouse gut development and differentiation, and carnitine deficiency leads to increased apoptosis of enterocytes, villous atrophy, inflammation and gut injury. Carnitine deficiency led to increased expression of CD45-B220^+^ lymphocytes with increased production of basal and anti-CD3-stimulated pro-inflammatory cytokines in immune cells [13]. L-carnitine administered prior to the irradiation reduced the severity of intestinal mucosal damage as treatment with L-carnitine decreased the serum MCP-1 and IFN-γ levels considerably [45].

This work may denote a possible association between GI manifestations and the reduced levels of serum carnitine and plasma DHA in autistic patients as a result of the decrease of their anti-inflammatory effects. It is not evident if GI manifestations are mere association or consequence to reduced PUFAs and carnitine levels in these patients. Further investigations are recommended, with a larger subject population, to determine the possible pathogenic role of the deficiency of PUFAs and carnitine levels in the occurrence of GI manifestations in autistic patients.

## Conclusions

Some autistic children have reduced levels of plasma PUFAs and serum carnitine which may be associated to GI manifestations in these patients. However, these data should be treated with caution until further investigations are performed, with a larger subject population, to determine whether the occurrence of GI manifestations is a mere association or a consequence to reduced plasma PUFAs and serum carnitine levels in autistic patients. Studies looking at a potential role of PUFAs and carnitine in treatment of autism and amelioration of GI problems are recommended.

## Abbreviations

AA: Arachidonic acid
ALA: α-linolenic acid
CARS: Childhood Autism Rating Scale
DHA: Docosahexaenoic acid
EPA: Eicosapentaenoic acid
GI: Gastrointestinal
IQR: Interquartile range
LA: Linoleic acid
PUFAs: Polyunsaturated fatty acids
SCFAs: Short-chain fatty acids
